# Leveraging deep survival models to predict quality of care risk in diverse hospital readmissions

**DOI:** 10.1038/s41598-023-37477-3

**Published:** 2023-06-28

**Authors:** Nhat Quang Tran, Gautam Goel, Nirmala Pudota, Michael Suesserman, John Helms, Daniel Lasaga, Dan Olson, Edward Bowen, Sanmitra Bhattacharya

**Affiliations:** 1 AI Center of Excellence, Deloitte & Touche LLP, New York, USA; 2 AI Center of Excellence, Deloitte & Touche Assurance & Enterprise Risk Services India Private Limited, Hyderabad, India; 3 Program Integrity, Deloitte & Touche LLP, New York, USA

**Keywords:** Computer science, Health services, Computational science

## Abstract

Hospital readmissions rate is reportedly high and has caused huge financial burden on health care systems in many countries. It is viewed as an important indicator of health care providers’ quality of care. We examine the use of machine learning-based survival analysis to assess quality of care risk in hospital readmissions. This study applies various survival models to explore the risk of hospital readmissions given patient demographics and their respective hospital discharges extracted from a health care claims dataset. We explore advanced feature representation techniques such as BioBERT and Node2Vec to encode high-dimensional diagnosis code features. To our knowledge, this study is the first to apply deep-learning based survival-analysis models for predicting hospital readmission risk agnostic of specific medical conditions and a fixed window for readmission. We found that modeling the time from discharge date to readmission date as a Weibull distribution as in the SparseDeepWeiSurv model yields the best discriminative power and calibration. In addition, embedding representations of the diagnosis codes do not contribute to improvement in model performance. We find dependency of each model’s performance on the time point at which it is evaluated. This time dependency of the models’ performance on the health care claims data may necessitate a different choice of model in quality of care issue detection at different points in time. We show the effectiveness of deep-learning based survival-analysis models in estimating the quality of care risk in hospital readmissions.

## Introduction

### Background

#### Hospital readmission rate is high

The rate of readmissions has been reported to be relatively high globally^1–4^. A study of hospital discharges of 12 million Medicare beneficiaries from 2 years of claims data reveals that nearly 20% of patients are readmitted within 30 days of discharge, 34% within 90 days, and over 56% within a year^1^. An analysis of 1306 inpatients aged 75 and older shows early unplanned readmissions happen at a rate of 14.2%^2^. Among patients with congestive heart failure, the readmission rate can be as high as 44% in 6 months^3^. This patient population is also among the highest early readmission rate in Canada and the United States (US)^4^.

Hospital readmissions can place a huge financial burden on health care systems. In 2004, unplanned hospital readmissions accounted for USD 17.4 billion of the USD 102.6 billion paid by Medicare to hospitals^1^. In 2011, around 3.3 million adults in the US were readmitted within 30 days, associated with about USD 41.3 billion in hospital costs^5^. Canadian Institute for Health Information (CIHI) estimated a CAD 1.8 billion cost incurred by readmissions to acute care during an 11-month study period (excluding physician fees for services), accounting for 11% of total inpatient care costs^6^.

#### Hospital readmission rate as an indicator of quality of care

In addition to incurring financial burdens on the health care system, hospital readmissions have also been viewed as red flags in hospitals’ quality of care^7^. CIHI reports that between 9 and 59% of readmissions may be prevented by improving patient education, discharge planning, appropriately scheduling follow-up appointments, and conducting follow-up communications^8^. Reasons that may directly indicate quality of care, such as length of stay, have also been shown to have a direct contribution to hospital readmissions^1^. Boutwell and Hwu^9^ suggests that for the patients with heart failure subgroup, the hospital readmission rate can be reduced by improved care, patient education, team management, and end-of-life care planning. In the US, the Centers for Medicare and Medicaid Services (CMS) established penalties for hospitals with high 30-day readmission rate by reducing the payment for readmitted patients^10^. In 2019, under the penalties program of CMS’s Hospital Readmission Reduction Program, 82% of hospitals were penalized for having excess readmissions^11^. CMS includes the following six medical conditions to evaluate unplanned readmissions in the program:

- Acute myocardial infarction (AMI).
- Chronic obstructive pulmonary disease (COPD).
- Heart failure (HF).
- Pneumonia.
- Coronary artery bypass graft (CABG) surgery.
- Elective primary total hip arthroplasty and/or total knee arthroplasty (THA/TKA).

Besides the US, the United Kingdom (UK), Denmark, and Germany have also introduced policies, financial or non-financial, to monitor hospital readmission rates^12^.

### Objective

Since early hospital readmissions have been established as a measure to control for quality of care of medical services, our goal is to understand the risk of hospital readmissions given the information related to patients and their respective hospital discharges in Medicare/Medicaid claims data. Most previous studies have focused on the prediction of hospital readmission risk for comparisons among hospitals or for facilitating targeted interventions during or after hospital discharges^13^. These studies aim to predict the probability that a patient is readmitted within a specific time frame (usually 30 or 90 days), often using simple rule-based models such as the LACE index^14^ or the HOSPITAL score^15^. A literature review by Ref.^16^ reveals that 52 out of 76 studies use logistic regression to predict the likelihood of hospital readmission. Some other methods explored in prior research include support vector machines^17–21^, decision tree-based techniques^17,22^, Bayesian methods^22^, and ensemble methods (e.g., boosting, bagging and random forest^4,17,18,20–24^). The majority of these studies structure the problem of hospital readmission risk prediction as a binary classification problem—whether a hospital discharge results in readmission within a certain number of days.

Another line of research is to learn a distribution of hospital readmission risk over time since an initial hospital discharge. For any time after discharge, these models predict the probability of hospital readmission occurring at or before the actual readmission time using survival analysis (or time-to-event analysis). The most commonly used survival model is the Cox Proportional Hazards model (Cox PH)^1,18,21,25–27^. A few studies have also implemented Random Survival Forest model, which decorrelates individual trees in the tree-based ensemble^27,28^. More recently, studies have shown that neural networks can improve the performance of traditional survival analysis models^29–35^. For example, DeepWeiSurv^30,31^ uses a multi-task learning neural network on the Molecular Taxonomy of Breast Cancer International Consortium (METABRIC) dataset (a UK-Canada project which tries to classify breast tumors into subcategories) and the Surveillance, Epidemiology, and End Results (SEER) dataset (which provides information on cancer statistics) to show that neural network based survival models outperform traditional survival models such as Cox PH. Similar to DeepWeiSurv, another fully parametric approach is Deep Survival Machines (DSM)^33^. DSM does not require constant proportional hazards assumption of the underlying survival distribution for time-to-event prediction. In contrast to DeepWeiSurv which learns the Weibull parameters and mixture coefficients from multi-layer perceptions following the latent representations, DSM learns these parameters directly from the latent representation.

Apart from restricting the analyses to a certain time frame after a hospital discharge, most studies focus on only one or a small set of medical conditions or diagnoses. Major conditions and diagnoses include heart failure^4,18,18,19,24–26,36^, acute myocardial infarction^23,36,37^, pneumonia^36,38^, diabetes^22^, and chronic obstructive pulmonary (COPD)^20^.

### Significance

To our knowledge, our study is the first to apply neural network-based survival-analysis models to predict hospital readmission risk from health care claims data, agnostic of specific medical conditions and a fixed window for readmission. There are several benefits to taking this approach in the context of quality of care. First, it allows us to identify quality of care issues for patients with *any* medical condition. This is especially important for claims data where patient populations are not segregated based on their diagnosis. Second, it gives us the probability of readmission within *any* time frame, making readmissions after the traditional 30-day or 90-day time frames also eligible for inspection on potential quality of care issues. While 30-day or 90-day time frames may be critical for policy compliance, these arbitrary time frames are not amenable to the diversity of medical conditions and corresponding discharge/ readmission times we consider in our study.

## Materials and methods

A survival analysis framework is adopted in this study where a distribution over time to an event from a particular starting point is estimated. In our case, this *time-to-event* is the time elapsed between a hospital discharge and subsequent readmission for *similar* medical conditions. In survival analysis, typically, *censored* data needs to be handled. Censoring happens when a study subject is not being monitored or observed at a particular point in time (also known as censored time), and the occurrence of an event after the censored time is unknown. The two primary reasons for a data point to be censored in survival analysis are (1) a subject withdraws from the study so their information beyond the withdrawal time is unavailable, and (2) after a pre-specified cut-off time a subject is not monitored and hence survival data is not collected. In our study, censoring happens primarily due to the latter reason as we do not consider hospital readmissions after $T=1095$ days (3 years). While there may be other possible reasons for censoring, such as a patient changes their health insurance program and can no longer be tracked, or a patient expires at home, such events cannot be observed and hence not considered in our study.

### Problem statement

In this section we formalize how we apply survival analysis to our data:

- A covariate matrix ${\varvec{X}}\in {\mathbb{R}}^{N\times d}$ that represents $d$-dimension feature vectors of $N$ hospital discharges. ${x}_{n}={\varvec{X}}\left[n\right]\left[:\right]$ is the feature vector for the $n$-th discharge in the dataset.
- The time elapsed ${t}_{n}\in \mathbb{R}$ since the $n$-th discharge to either a readmission or a censored time.
- Censoring variable ${\delta }_{n}\in \{\mathrm{0,1}\}$ that indicates whether a readmission occurs at time ${t}_{n}$ after the $n$-th discharge or it is censored at ${t}_{n}$.

Also denote ${T}_{n}$ as the actual time of readmission following the $n$-th discharge (${T}_{n}\equiv {t}_{n}$ if ${\updelta }_{n}=1$).

The goal is to estimate the distribution

1 $$\begin{array}{c}f\left(t|{x}_{n}\right)\sim Pr\left({T}_{n}=t|{x}_{n},{t}_{n},\delta \right).\end{array}$$

Most survival models do not learn $f(t)$ directly. For example, the Cox PH model and its extensions (introduced in Models section) learn the hazard function:

2 $$\begin{array}{c}\lambda \left(t\right)=-\frac{d}{dt}{\text{log}}S\left(t\right),\end{array}$$

where $S\left(t\right)$ is the survival function:

3 $$\begin{array}{c}S\left(t\right)={\text{P}}{\text{r}}\left({T}_{n}\ge t\right).\end{array}$$

The hazard function is the instantaneous rate of occurrence of the event at a particular time point and we can derive the desired density function from it.

### Study data

We conducted this study on 222,175 redacted and anonymized inpatient medical claims from state Medicare programs. The dataset was redacted and anonymized following the Safe Harbor method, Section 164.514(b)(2) of the HIPAA Privacy Rule.

#### Data overview

A claim submitted to a Medicare program typically includes the following information:

- *Claim number* a distinct identifier of a claim.
- *Diagnosis codes* encoded using the International Classification of Diseases (ICD-10)^39^, a standardized system used to encode clinical terms. A claim contains at least a primary diagnosis code, and optionally secondary and tertiary diagnosis codes. An ICD-10 code consists of up to 7 characters that distinctly identify a medical condition. The first three characters represent the general diagnosis, and the other characters represent more specific categories. Examples of a hierarchical break-down for general diagnosis codes I05 and I06 are shown in Table 1.
  When considering readmissions, we only analyze the first three digits of the ICD-10 codes, which represent the general category of the diagnoses. This helps us generalize our model by considering related diagnosis for which a patient may be readmitted.
- *Procedure codes* represented by Current Procedural Terminology (CPT) codes^40^, encodes procedures performed by health care providers.
- *Provider ID* a unique identifier that represents the health care provider that submitted the claim, as used in National Provider Identifier (NPI)^41^ registry.
- *Patient demographics* patient sex and age.
- *Admittance date and discharge date* the dates when a patient is admitted and discharged.
- *Billed amount* the total amount billed for services rendered by the health care provider.

**Table 1:** Hierarchical breakdown of diagnosis codes.

Code	Description
I05-I09	Chronic rheumatic heart diseases
I05	Rheumatic mitral valve diseases
I05.0	Rheumatic mitral stenosis
I05.1	Rheumatic mitral insufficiency
I06	Rheumatic aortic valve diseases
I06.0	Rheumatic aortic stenosis
I06.1	Rheumatic aortic insufficiency

#### Data pre-processing and representation

From a dataset of over 8 million claims, we filter for only inpatient claims, where a patient gets admitted to a hospital, and claims that have a positive paid amount. We construct two subsets: *readmission claims* and *non-readmission claims.* The readmission subset includes initial admissions and the subsequent readmissions of the same patient with the same general diagnosis codes. The non-readmission subset includes admissions without any subsequent readmissions.

**Figure 1:**
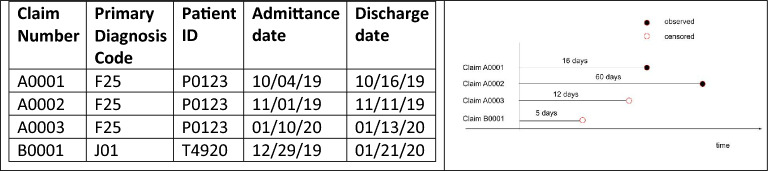
An example of data for four claims and how they are represented for survival analysis. *Left* Patient P0123 has three admissions (associated with three claim numbers) for the same diagnosis coded as F25 (schizoaffective disorders) and Patient T4920 only has one admission. *Right* For each claim number, the *time-to-event* is the time from the discharge date to the admittance date of the subsequent admission. For example, Claim A0001’s time-to-event is 16 days (10/16/19–11/01/19). If an admission does not have any subsequent readmission (A0003 and B0001 in this case), the *time-to-event* is the time from the discharge date to the latest date recorded in the dataset 01/26/2020, and its censoring indicator is marked as *censored.* For example, B0001’s time-to-event is 5 days (01/21/20–1/26/20) and is marked as censored. This is interpreted as the time-to-event for this discharge is *at least* 5 days, but we do not know when exactly the readmission will happen after 5 days (could be indefinitely long). A0001–0003 claims are in the readmission subset. B0001 is in the non-readmission subset. All claims in the non-readmission subset are censored. The last claim in each readmission chain in the readmission subset (e.g., claim A0003) is censored. The other claims are *observed*.

To represent the dataset in a way that conforms to the structure of the survival data, we define a *time-to-event* and *censoring indicator* for each admission and readmission. Figure 1 illustrates how the data is represented. The time-to-event of each admission is the time (in days) from the discharge date of that admission to the admittance date of the next readmission. For admissions that are not followed by a readmission, the exact time-to-event is unknown, so we use the time from the discharge date to the last recorded date in the data (01/26/2020). These admissions are said to be *censored.*

Based on patient ID numbers, we split 222,175 total claims into training, validation, and test sets. Because of the severe class imbalance (readmission cases comprise of only 13% of the entire dataset), we downsample the training subset by first sampling one claim per patient in the non-readmitted set and then subsampling from these claims so that the size of the non-readmitted and that of the readmitted datasets are equal. Table 2 shows the observed vs. censored ratio in the training, validation, and test datasets. Supplementary Appendix A shows the dimension of each feature and the corresponding summary statistics (for applicable variables) for $n=\mathrm{222,175}$ claims.

**Table 2:** Prevalence of observed cases in datasets.

	# Observed/total (%)
Training	27,769/91,163 (30.46%)
Validation	6914/22,805 (30.31%)
Test	8747/108,207 (8.08%)

#### Feature engineering

We use the following features in our survival analysis.

- *Patient age* Age bucketed into five categories: 0–17 years, 18–38 years, 39–59 years, 60–80 years, and greater than 80 years. We one-hot encode this feature.
- *Patient sex* patient sex is binarized into 0 (female) and 1 (male). No non-binary sex is present in the data.
- *Specialty code* this code represents the specialty^42^ of the respective health care provider and is one-hot encoded. Empty code is represented as the ‘UNK’ (unknown) category.
- *Length of hospital stay* the difference in days between the discharge date and the admittance date. Claims with zero length of stay along with those within the same readmission chain with these claims are removed.
- *Diagnosis code* each claim number has at least one and at most three diagnosis codes. The primary code is always present. We only consider the first three digits of the codes as the general category of the diagnosis. For each claim number (a data point), we collect all the general diagnosis codes and multi-hot encode them. We do not consider codes that appear less than 100 times in the entire dataset and code them into an *Other* category.

Besides the multi-hot encoding approaches for feature representation, we experimented with word embedding and graph embedding models for feature representation of the diagnosis codes. For word embedding, we use the embeddings (dimension: 200) obtained from the BioWordVec model^43^ trained on the string descriptions of the diagnosis codes. For graph embedding (dimension: 256), we use the Node2Vec model^44^ trained on five million claims from the same dataset in this study, with the proxy task of link prediction (predicting whether or not a link exists between a pair of nodes).

While other features from the claims data could be viewed as relevant to readmission modeling, similar to previous studies^45,46^, we focus on patient demographics and diagnosis as key indicators in estimating the likelihood of readmission. All the features listed above are concatenated into a vector representing features for the respective claim.

### Models

We conduct a series of experiments to evaluate five survival analysis models (detailed in the next section) on our data.

#### Cox proportional hazards (Cox PH)

Cox PH is one of the most common regression models and baseline models in survival analysis^1,21,25–27,47^. Cox PH assumes linearity to model the hazard function:

4 $$\begin{array}{c}\lambda \left(t|{x}_{n}\right)={\lambda }_{0}\left(t\right){e}^{\beta \cdot {x}_{n}}.\end{array}$$

Notice that ${\uplambda }_{0}\left(t\right)$, the *baseline hazard*, depends on time but does *not* depend on the covariates ${x}_{n}$. It describes the risk of readmission when ${x}_{n}=0$, and the exact risk level of each discharge is scaled by the exponential that depends on ${x}_{n}$ (*proportional hazard assumption*). For this reason, the value $\mathit{exp}\{\beta \cdot {x}_{n}\}$ (also known as the *hazard ratio*) is characteristic of an individual’s relative risk level compared to other individuals. Cox PH can be viewed as a regression model, which tries to estimate $\upbeta$ to maximize the partial likelihood of data:

5 $$\begin{array}{c}L\left(\beta \right)=\prod_{n:{\delta }_{n}=1}{L}_{n}\left(\beta \right),\end{array}$$

where

6 $$\begin{array}{c}{L}_{n}\left(\beta \right)=\frac{\lambda \left({t}_{n}|{x}_{n}\right)}{{\sum }_{m:{t}_{m}\ge {t}_{n}}\lambda \left({t}_{n}|{x}_{m}\right)}.\end{array}$$

#### C-mix model

Besides Cox PH, we also include the C-mix model in our experiment, which is reported to outperform other survival models experimented in Ref.^21^, a study on predictive models for hospital readmissions following vaso-occlusive crisis (VOC). C-Mix was originally designed to identify subgroups in the data with varying risk levels. It models the density of time-to-event as a mixture of Weibull distributions. In the case of a two-component mixture, the density is:

7 $$\begin{array}{c}f\left(t|{x}_{n}\right)={\pi }_{0}\left({x}_{n};{\beta }_{0}\right){f}_{0}\left(t;{\alpha }_{0}\right)+{\pi }_{1}\left({x}_{n};{\beta }_{1}\right){f}_{1}\left(t;{\alpha }_{1}\right),\end{array}$$

where

$$\uppi \left(x;{\upbeta }_{k}\right)=\frac{{e}^{x\cdot {\upbeta }_{k}}}{{\sum }_{k=0}^{1}{e}^{x\cdot {\upbeta }_{k}}},$$

where ${\pi }_{0}(\cdot )$ and ${\pi }_{1}(\cdot )$ are the weights for the components, ${f}_{1}$ and ${f}_{2}$ are two Weibull distributions parameterized by vectors ${\alpha }_{1}$ and ${\alpha }_{2}$, respectively, and:

8 $$\begin{array}{c}{\pi }_{i}\left(x;{\beta }_{i}\right)={\text{e}}{\text{x}}{\text{p}}\left(x\cdot {\beta }_{i}\right)/\left(\sum_{j\in \left\{\mathrm{1,2}\right\}}{\text{exp}}\left(x\cdot {\beta }_{j}\right)\right),i\in \mathrm{1,2}.\end{array}$$

The two components ${f}_{0}$ and ${f}_{1}$ in the mixture can be viewed as representing two subgroups: the high risk and the low risk groups, respectively. Then, ${\pi }_{0}({x}_{n};{\beta }_{0})$ can be interpreted as the risk level. The parameters are estimated by minimizing the negative log likelihood of data.

#### DeepSurv

DeepSurv^29^ is an extension of Cox PH as it uses a neural network to model the hazard function:

9 $$\begin{array}{c}\lambda \left(t|{x}_{n}\right)={\lambda }_{0}\left(t\right){e}^{{h}_{\theta }\left({x}_{n}\right)},\end{array}$$

where $${h}_{\uptheta }(\cdot )$$ is a multilayer perceptron (MLP) with weights $$\uptheta$$. Notice that this is almost identical to Eq. ($$1)$$ except that the relationship with the covariates is modeled by an MLP instead of a linear function. DeepSurv is optimized by minimizing the negative log partial likelihood with regularization:

10 $$\begin{array}{c}{L}_{\text{DeepSurv}}=-\frac{1}{{N}_{{\lambda }_{n}=1}}{\sum }_{n:{\delta }_{n}=1}\left({h}_{\theta }\left({x}_{n}\right)-\mathit{log}{\sum }_{j:{t}_{j}\ge {t}_{i}}{e}^{{h}_{\theta }\left({x}_{j}\right)}\right)+\lambda {\Vert \theta \Vert }_{2}^{2},\end{array}$$

where $\uplambda$ is the regularization strength.

#### Sparse DeepWeiSurv

DeepWeiSurv^30^ models the density over time to readmission as a mixture ${f}_{W}$ of $K$ Weibull distributions:

11 $$\begin{array}{c}f\left(t|{x}_{n},{\theta }_{n}\right)={\sum }_{k=1}^{K}{\alpha }_{k}\left({x}_{n}\right){f}_{{\beta }_{k}\left({x}_{n}\right), {\eta }_{k}\left({x}_{n}\right)},\end{array}$$

where ${\mathrm{\alpha }}_{k}$ is the weight, and ${\upbeta }_{k}$ and ${\upeta }_{k}$ are the shape and scale parameters of the $k$-th Weibull component in the mixture (note that these parameters depend on the covariates ${x}_{n}$). The goal of DeepWeiSurv is to learn $\mathrm{\alpha }\in {\mathbb{R}}^{K},\upbeta \in {\mathbb{R}}^{K},\upeta \in {\mathbb{R}}^{K}$ from each ${x}_{n}$. DeepWeiSurv adopts a multi-task learning approach: there is a common sublayer ${f}_{DWS}$ that is a MLP that learns a representation of ${x}_{n}$:

12 $$\begin{array}{c}{{z}_{n}=f}_{DWS}\left({x}_{n}\right).\end{array}$$

After that DeepWeiSurv learns two MLP’s ${f}_{1}$ and ${f}_{2}$:

13 $$\begin{array}{c}\alpha \left({x}_{n}\right)={f}_{1}\left({z}_{n}\right),\end{array}$$

14 $$\begin{array}{c}\beta \left({x}_{n}\right),\eta \left({x}_{n}\right)={f}_{2}\left({z}_{n}\right).\end{array}$$

SparseDeepWeiSurv^31^ extends DeepWeiSurv by incorporating a sparsing layer in ${f}_{1}$ to learn the number of components in the mixture. The model is optimized by minimizing the negative log likelihood. SpraseDeepWeiSurv outperforms DeepWeiSurv across five real-world datasets.

#### Deep cox mixture (DCM)

DCM^34^ fuses the Cox PH and DeepSurv to obtain a deep learning model that learns a mixture of Cox PH to model individual time-to-event distribution. It assumes there are latent groups and within each group, the proportional hazard assumption holds. In each Cox group of the mixture, DCM fits the hazard ratios using deep neural networks and the baseline hazard for each mixture component non-parametrically. It is reported to have a state-of-the-art performance on time-to-event regression tasks on survival data on mortality (e.g., METABRIC, SEER^30^).

### Evaluation metrics

As pointed out by Ref.^34^, most studies on survival models evaluate them using the relative ranking of the predictions of the risk level such as the concordance index (C-index). However, these metrics disregard the absolute values of the probability predictions, while these probabilities are directly used when detecting quality of care issues. The set of metrics that only depend on the ranking in terms of risk level of data points measure models’ *discriminative power,* while those factoring in the actual predicted probabilities of readmission measure *calibration.* Following^34^, we assess the statistical models on both aspects with the following 4 metrics. All metrics are time-dependent. In this study, we evaluate the metrics at the time points that are the 25th, 50th and 75th percentile of event times in our dataset.

#### Time-dependent concordance index (discrimination metric)

The C-index measures the proportion of all eligible pairs of observations that are correctly ranked in terms of risk. The time-dependent concordance index restricts these comparisons to instances that occur within a certain time frame.

15 $$\begin{array}{c}C\left({{\varvec{t}}}_{0}\right)=\frac{{\sum }_{{\varvec{i}},{\varvec{j}}}{\delta }_{{\varvec{i}}}1\left({{\varvec{t}}}_{{\varvec{i}}}<{{\varvec{t}}}_{{\varvec{j}}}\right)1\left({{\varvec{t}}}_{{\varvec{i}}}\le {{\varvec{t}}}_{0}\right)1\left({\varvec{\lambda}}\left({{\varvec{t}}}_{{\varvec{i}}}|{{\varvec{x}}}_{{\varvec{i}}}\right)>{\varvec{\lambda}}\left({{\varvec{t}}}_{{\varvec{j}}}|{{\varvec{x}}}_{{\varvec{j}}}\right)\right)}{{\sum }_{{\varvec{i}},{\varvec{j}}}{\delta }_{{\varvec{i}}}1\left({{\varvec{t}}}_{{\varvec{i}}}<{{\varvec{t}}}_{{\varvec{j}}}\right)1\left({{\varvec{t}}}_{{\varvec{i}}}\le {{\varvec{t}}}_{0}\right)}.\end{array}$$

#### Area under the receiver operation characteristic curve (AUC) (discrimination metric)

At any point in time ${t}_{0}$, we can retrieve a binary label for any data point that indicates whether the readmission has happened by that time. Using $\uplambda \left({t}_{0}|{x}_{i}\right)$ to score an example $i$, we can compute the AUC as in a typical binary classification problem (using logistic regression for example).

#### Expected calibration error (ECE) (calibration metric)

ECE is the average absolute difference between the observed and the predicted readmission rate, given the predicted readmission rate. Let the predicted readmission rate at time ${t}_{0}$ be $R\left({t}_{0}|{x}_{i}\right)=\widehat{P}\left({t}_{i}<{t}_{0}|{x}_{i}\right)$, then

16 $$\begin{array}{c}ECE\left({t}_{0}\right)=E\left(\left|R\left({t}_{0}\right)-P\left(T>{t}_{0}|R\left({t}_{0}\right)\right)\right|\right).\end{array}$$

We can estimate $ECE\left({t}_{0}\right)$ by bucketing $R\left({t}_{0}\right).$

#### Brier score (dual metric)

Brier score computes the mean squared error that quantifies the deviation of the predicted readmission rate within a time frame from the censoring indicator.

17 $$\begin{array}{c}BR\left({t}_{0}\right)=\frac{{\sum }_{i}1\left({t}_{i}>{t}_{0}\right){\left(0-R\left({t}_{0}|{x}_{i}\right)\right)}^{2}+1\left({t}_{i}\le {t}_{0}\right){\delta }_{i}{\left(1-R\left({t}_{0}|{x}_{i}\right)\right)}^{2}}{{\sum }_{i}1\left({t}_{i}>{t}_{0}\right)+1\left({t}_{i}\le {t}_{0}\right){\delta }_{i}}.\end{array}$$

For model tuning and validation, we use a vanilla (non-time-dependent) version of the concordance index, which is traditionally used to evaluate survival analysis models and computed as:

18 $$\begin{array}{c}C=\frac{{\sum }_{i,j}1\left({t}_{j}<{t}_{i}\right)1\left({\lambda }_{j}>{\lambda }_{i}\right){\delta }_{j}}{{\sum }_{i,j}1\left({t}_{j}<{t}_{i}\right){\delta }_{j}}.\end{array}$$

## Results

### Experiment setting

For deep models (DeepSurv, SparseDeepWeiSurv, and DCM), we tune the models’ hyper-parameters based on the computed concordance index on the validation subset. Further training details are provided in Supplementary Appendix B.

### Results

For each of the five models, following the approach in Ref.^34^, we compute the four evaluation metrics at three time-quantiles, 25th, 50th and 75th ones. The 25th, 50th and 75th time-quantiles correspond to readmission time frames of 17 days, 49 days, and 123 days. The metrics are reported in Table 3 for the test dataset.

**Table 3:** AUC, C-index, Brier score, and ECE computed at the 25th, 50th and 75th percentiles for the five models on the test set.

	AUC	C-index	Brier score	ECE
25th percentile				
Cox PH	0.817	0.812	0.021	0.037
C-mix	0.801	0.795	0.021	0.020
DeepSurv	0.801	0.795	0.022	0.037
DCM	**0.822**	**0.817**	0.022	0.034
SparseDeepWeiSurv	0.821	0.815	**0.020**	**0.007**
50th percentile				
Cox PH	0.832	**0.827**	0.043	0.076
C-mix	0.817	0.809	0.041	0.060
DeepSurv	0.814	0.807	0.045	0.077
DCM	0.832	0.825	0.043	0.071
SparseDeepWeiSurv	**0.834**	**0.827**	**0.036**	**0.012**
75th percentile				
Cox PH	0.839	0.827	0.067	0.118
C-mix	0.826	0.814	0.073	0.121
DeepSurv	0.819	0.808	0.072	0.121
DCM	0.829	0.818	0.072	0.114
SparseDeepWeiSurv	**0.841**	**0.829**	**0.053**	**0.040**

The diagnosis codes are multi-hot encoded.

The best performing value for each evaluation metric is highlighted in bold.

For a short window (e.g., 25th percentile), the DCM model has the highest discriminative power, with AUC of 0.822 and C-index of 0.817, closely matched by SparseDeepWeiSurv, with AUC of 0.821 and C-index of 0.815. SparseDeepWeiSurv, on the other hand, is the best calibrated with the lowest Brier score and ECE. Notably, its ECE at 0.007 is 79% lower than the second lowest ECE of 0.034 achieved by DCM.

For larger time windows (i.e., 50th and 75th percentiles), SparseDeepWeiSurv outperforms other models in both calibration and discrimination with best performance across all four metrics. For these larger time windows, Cox PH closely matches SparseDeepWeiSurv on discrimination metrics (e.g., for 50th percentile window, both Cox PH and SparseDeepWeiSurv have a C-index of 0.827). With respect to ECE, as with a smaller percentile window, the gap between the performance of SparseDeepWeiSurv is large (80% and 69% lower than the second-lowest ECE in 50th percentile and 75th percentile windows, respectively). DCM has lower discriminative power but better calibration compared to Cox PH. C-mix and DeepSurv models consistently have the lowest performance across almost all metrics and percentiles, with the exception of C-mix’s ECE of 0.060 at 50th percentile, where it achieves the second best calibration score.

As discussed in “Feature engineering” section, we also experimented with word and graph embedding based feature representation of the diagnosis codes. The results of using these embeddings are reported in Tables 4 and 5.

**Table 4:** AUC, C-index, Brier score, and ECE computed at the 25th, 50th and 75th percentiles for the five models on the test set.

	AUC	C-index	Brier score	ECE
25th percentile				
Cox PH	0.800	0.795	0.021	0.037
C-mix	0.778	0.774	0.020	0.022
DeepSurv	0.801	0.795	0.022	0.035
DCM	0.801	0.797	0.021	0.034
SparseDeepWeiSurv	**0.805**	**0.799**	**0.019**	**0.004**
50th percentile				
Cox PH	0.812	0.805	0.043	0.076
C-mix	0.787	0.781	0.042	0.066
DeepSurv	0.813	0.806	0.045	0.035
DCM	0.810	0.804	0.042	0.072
SparseDeepWeiSurv	**0.818**	**0.811**	**0.035**	**0.008**
75th percentile				
Cox PH	0.820	0.809	0.069	0.117
C-mix	0.797	0.787	0.075	0.128
DeepSurv	0.818	0.807	0.074	0.116
DCM	0.800	0.789	0.073	0.124
SparseDeepWeiSurv	**0.824**	**0.813**	**0.053**	**0.043**

The diagnosis codes are embedded using word embedding.

The best performing value for each evaluation metric is highlighted in bold.

**Table 5:** AUC, C-index, Brier score, and ECE computed at the 25th, 50th and 75th percentiles for the five models on the test set.

	AUC	C-index	Brier score	ECE
25th percentile				
Cox PH	0.809	0.805	0.021	0.036
C-mix	0.796	0.793	**0.020**	0.022
DeepSurv	0.813	0.807	0.021	0.035
DCM	**0.818**	**0.814**	0.021	0.036
SparseDeepWeiSurv	0.810	0.804	**0.020**	**0.019**
50th percentile				
Cox PH	0.823	0.815	0.043	0.074
C-mi	0.806	0.799	0.042	0.064
DeepSurv	0.827	0.820	0.042	0.074
DCM	**0.828**	**0.821**	0.043	0.075
SparseDeepWeiSurv	0.823	0.816	**0.040**	**0.052**
75th percentile				
Cox PH	0.831	0.819	0.069	0.115
C-mix	0.812	0.801	0.074	0.124
DeepSurv	**0.836**	**0.824**	0.067	0.115
DCM	0.821	0.810	0.073	0.120
SparseDeepWeiSurv	0.828	0.817	**0.066**	**0.095**

The diagnosis codes are embedded using graph embedding.

The best performing value for each evaluation metric is highlighted in bold.

Using word embeddings of the diagnosis codes, we observe a minor improvement in the best calibration score ECE. For example, the best ECE at 25th percentile when using multi-hot encoded diagnosis codes is by SparseDeepWeiSurv (0.007 ECE), and the corresponding figure for word embedding is 0.004. However, with metrics at 75th percentile, the ECE performs worse than in the multi-hot encoding experiments (e.g., for SparseDeepWeiSurv, performance is worsened from 0.040 to 0.043). Using graph embeddings, we observe a decrease in performance across all metrics and models. Overall, we do not see significant improvement in model performance when incorporating advanced embedding techniques for embedding health care diagnosis codes.

Finally, we conduct an experiment with a 30-day readmission time frame. This time frame is commonly used in the existing literature on hospital readmission analysis and makes our finding comparable to other studies. Since word or graph embeddings of diagnosis codes do not improve model performance, we conduct this experiment with multi-hot encoding of diagnosis codes. Results in Table 6 show that the DCM model has the highest discriminative power with AUC and C-index scores of 0.836 and 0.831, respectively. SparseDeepWeiSurv is the best calibrated model with the lowest Brier score and ECE of 0.029 and 0.009, respectively.

**Table 6:** AUC, C-index, Brier score, and ECE computed for 30-day readmission for the five models on the test set.

30 day				
	AUC	C-index	Brier Score	ECE
Cox PH	0.827	0.821	0.032	0.056
C-mix	0.811	0.805	0.031	0.036
DeepSurv	0.811	0.805	0.033	0.056
DCM	**0.836**	**0.831**	0.032	0.054
SparseDeepWeiSurv	0.830	0.824	**0.029**	**0.009**

The diagnosis codes are multi-hot encoded.

The best performing value for each evaluation metric is highlighted in bold.

## Discussion

### Use different models at different time points

We see the dependency of each model’s performance on the time point at which it is evaluated. At a lower time point (e.g., at 25th percentile which is the 17-day time frame, and the 30-day time frame), DCM has the best discriminative power while SparseDeepWeiSurv is the best calibrated. For larger time frames (e.g., 50th and 75th percentiles), the performance of Cox PH improves to closely match SparseDeepWeiSurv for calibration, while the performance of DCM closely tracks the other two top performing methods, especially for the 50th time-quantile. This time dependency of the models’ performance on the health care claims data may necessitate a different choice of model in quality of care issue detection at different points in time. For example, suppose we are evaluating whether a readmission, which happens 100 days after its previous discharge, the choice of model used to evaluate the readmission should depend on which model has the best performance at $t=100$ days.

In our statistical tests for significance, the differences in performance of the DCM and SparseDeepWeiSurv are non-significant at the 25th and 50th percentiles of the discriminative metrics. In addition, SparseDeepWeiSurv outperforms DCM and other methods in calibration metrics. In our task of identifying unusually early readmissions, calibration plays a more important role than discrimination. The downstream decision is based on the predicted likelihood of readmission at and before the date a patient of interest is being readmitted to determine if the readmission falls out of the acceptable likelihood threshold. We also note that the simpler Cox PH has strong performance in terms of discriminative power, comparable with the much more complex model SparseDeepWeiSurv. Therefore, Cox PH may be favored in tasks that focus only on the ranking of patients’ readmission risk level.

DeepSurv, which removes the assumption of linearity in Cox PH and using a neural network to model the hazard function, consistently has worse performance than Cox PH across discrimination and calibration metrics. Further investigation is needed to understand why it underperforms CoX PH on our data.

There are some promising directions that we would like to explore as next steps. First, while our current approach is based solely on claims data, in future we would like to explore complementary data sources such as electronic health records through which we may be able to enrich our feature set to include lab results, radiology reports, etc. Second, since our models are built to allow for inference on patients with *any* medical condition (not restricted to one or a small set of medical conditions), we would like to investigate to what extent this relaxation compromises the models’ performance in either discriminative power or calibration. It is also important to know which of the four metrics are the most reliable and relevant for this problem of detecting quality of care issues through hospital readmission prediction.

### Limitations

Our study has several limitations. First, while we view quality of care issues through the lens of hospital readmissions (as do several prior studies (“Hospital readmission rate as an indicator of quality of care” section)), there are studies which have not found a strong link between readmissions and quality of care. For example, in Ref.^14^ the authors show that less than one-fifth of urgent readmissions were potentially avoidable based physician reviews of patient files in a prospective study. In contrast to most prior studies (including^12^), we focus on survival modeling to predict the likelihood of all-cause readmissions that may not be urgent (i.e. within 30 days) based on claims data. Second, the claims data we use in our experiments do not have an indicator of mortality, a confounding factor for survival analysis. The right-censoring of our data accounts for both mortality and no readmissions within 3 years following the initial admission. Third, our analysis is based on patients enrolled in the Medicare program (who are aged 65 years or over, younger people with disabilities, and people with End Stage Renal Disease) and our findings may not apply to readmissions data from other demographics.

## Conclusion

In this study, we frame the problem of identifying early readmissions following a discharge as a survival analysis problem, where we estimate the distribution over time to readmission after a discharge conditioned on the discharge’s covariates. We evaluate five models both on the discriminative power and the calibration. We observe that Cox PH and SparseDeepWeiSurv, the top performing models, have comparable discrimination ability; but SparseDeepWeiSurv, which models the time to readmission as a Weibull distribution, is the better calibrated. DeepSurv, which removes the linearity assumption of Cox PH and replaces it with a more complex relationship modeled by a neural network, has worse performance than Cox PH. DCM, an extension of DeepSurv, also generally performs worse than DeepSurv on our dataset. We also find that representing the diagnosis codes with advanced embedding methods such as those from Node2Vec and BioBERT does not improve and, in some cases, worsens model performance.

## Supplementary Information

Supplementary Information.

## Data Availability

The raw datasets analysed during the current study are not publicly available in full due to licensing and contractual restrictions, but synthetic sample datasets are available from the corresponding author on reasonable request. The source dataset was redacted and anonymized by a team who are specialized in this process, following the Safe Harbor method, Section 164.514(b)(2) of the HIPAA Privacy Rule. Deloitte holds contracts with various Medicare and Medicaid agencies through which it has access to this data. We cannot advice on conditions under which other researchers can access similar datasets. The CMS provides beneficiary-level health information to researchers which can be requested through https://www.cms.gov/research-statistics-data-and-systems/files-for-order/limiteddatasets. Implementations of the models we experimented with are the following: Cox PH https://lifelines.readthedocs.io/en/latest/index.html, C-mix https://github.com/SimonBussy/C-mix, DeepSurv https://github.com/jaredleekatzman/DeepSurv, DeepWeiSurv https://github.com/survml, DeepCoxMixture https://autonlab.org/auton-survival/.
