# Supplementary material for: Leveraging deep survival models to predict quality of care risk in diverse hospital readmissions
Source: Sci Rep. 2023 Jun 28;13:10479. doi: 10.1038/s41598-023-37477-3 (PMC10307854; doi:10.1038/s41598-023-37477-3)
Supplement: Supplementary file 1 — Supplementary Information. [file 41598_2023_37477_MOESM1_ESM.docx]

# APPENDIX A

| **Feature** | **Dimension** | **Mean** | **Median** | **Min** | **Max** |
| --- | --- | --- | --- | --- | --- |
| Age | 4 | NA | NA | NA | NA |
| Sex | 1 | NA | NA | NA | NA |
| Specialty code | 32 | NA | NA | NA | NA |
| Length of stay | 1 | 5.56 | 3.00 | 1.00 | 1104 |
| Diagnosis code | 713 | NA | NA | NA | NA |
| **Total** | **750** |  |  |  |  |

Table A. Dimension of data features

# APPENDIX B

*Details of models’ hyperparameters and hyperparameter tuning:* Implementations of the models we experimented with can be found in the following code repositories: Cox PH: <https://lifelines.readthedocs.io/en/latest/index.html> , C-mix: <https://github.com/SimonBussy/C-mix> , DeepSurv: <https://github.com/jaredleekatzman/DeepSurv> , DeepWeiSurv: code available from authors of [31], DeepCoxMixture: <https://autonlab.org/auton-survival/>.

Following the experimental design in [34], for all models, we choose Brier Score to be the base metric for training and model selection. All models were trained for a maximum of 100 epochs with early stopping with a patience of 3. We train DeepSurv for with Adam optimizer [40], and Scaled Exponential Linear Units (SELU) activation (as used in [27]), a batch size of 5,000, learning rate 7e-5, dropout rate 0.137, L2 regularization strength 0.068, learning rate decay rate 1e-4, and trained for 50 epochs. For SparseDeepWeiSurv, we use Rectified Linear Unit (ReLU) activation, Stochastic Gradient Descent for optimization, dropout rate 0.189, batch size 16, regularization strength 0.652, learning rate 7e-3, one component in the model’s mixture Weibull distribution, and trained for 17 epochs. For DCM, we used Adam optimizer, learning rate 3e-3, dropout rate 0.011, L2 regularization strength 1.380, learning rate decay rate 1e-5, and trained for 10 epochs. The range of the hyperparameters to search on during hyperparameter tuning is given in the table below.

| Hyperparameter | Value set |
| --- | --- |
| Learning rate | [1e-5, 1e-2] |
| Dropout rate | [0, 0.5] |
| L2 regularization strength | [0,5] |
| Learning rate decay rate | [1e-5, 1e-3] |
| Use of batch normalization | {Yes, No} |
| $\beta_{1}$ (of Adam optimizer) | [0.7, 0.9] |
| Batch size | {16, 128, 256} |

Table B-1 .DeepSurv and DCM hyper-parameter search range

| Hyperparameter | Value set |
| --- | --- |
| Learning rate | [1e-5, 1e-1] |
| Dropout rate | [0, 0.7] |
| L2 regularization strength | [0,1] |
| Learning rate decay rate | [1e-5, 1e-3] |
| Use of batch normalization | {Yes, No} |
| Maximum number of components in mixture | Up to 10 |
| Batch size | {16, 128, 256} |

Table B-2. SparseDeepWeiSurv hyper-parameter search range

**APPENDIX C**

Training times of DeepSurv, DCM and SparseDeepWeiSurv (with multi-hot encoded diagnosis codes) on an AWS SageMaker ml.p3.2xlarge instance (with 1 V100 GPU, 8 CPUs, and 61GB memory) are shown below. DeepWeiSurv takes the longest training time per epoch at 75.9 seconds.

- DeepSurv: 12.9 seconds per epoch, for 50 epochs the total time is 10.75 minutes
- DCM: 44.5 seconds per epoch, for 10 epochs total training time is 7.42 minutes
- SparseDeepWeiSurv: 75.9 seconds per epoch, for 17 epochs the total training time is 21.5 minutes
